# Dataset on safety and protective efficacy studies of COVID-19 vaccine candidates based on structurally modified plant virus in female hamsters

**DOI:** 10.1016/j.dib.2023.109158

**Published:** 2023-04-19

**Authors:** A.O. Kovalenko, E.M. Ryabchevskaya, E.A. Evtushenko, O.A. Kondakova, P.A. Ivanov, M.V. Arkhipenko, N.A. Nikitin, O.V. Karpova

**Affiliations:** Department of Virology, Faculty of Biology, Lomonosov Moscow State University, 1-12 Leninskie Gory, Moscow 119234, Russian Federation

**Keywords:** COVID-19, Vaccine, Structurally modified plant virus, Spherical particles, Preclinical study, Vaccine protective efficacy, Lung histological studies

## Abstract

This data article is related to the previous research, which addressed the development of a COVID-19 recombinant vaccine candidate. Here, we present the additional data in support of the safety and protective efficacy evaluation of two COVID-19 vaccine candidates based on the coronaviruses’ S protein fragments and a structurally modified plant virus – spherical particles. The effectiveness of the experimental vaccines was studied against the SARS-CoV-2 virus in an *in vivo* infection model in female Syrian hamsters. The body weight of vaccinated laboratory animals was monitored. The histological assessment data of the infected with the SARS-CoV-2 virus hamsters’ lungs are provided.

## Graphical abstract

**Figure:**
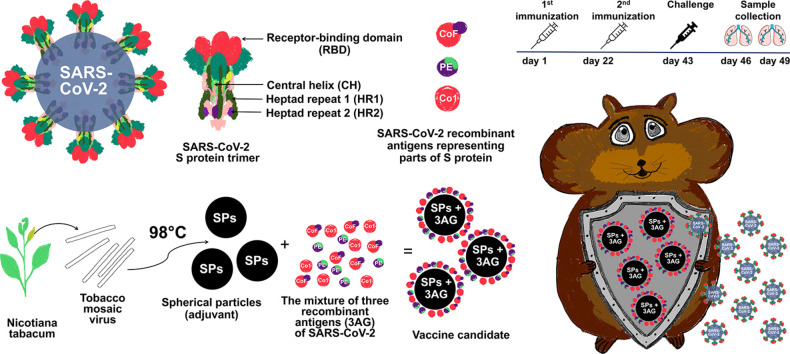

**Specifications Table**

**Table:** 

Subject	Biological sciences
Specific subject area	Human Vaccines, Plant Virus
Type of data	Tables, Images, Charts and Figures
How the data were acquired	The body weight of the vaccinated animals was monitored using an “MK-6.2-A21” electronic balance (MASSA-K, St. Petersburg, Russia). Histological preparations were analyzed using a Leica DM2000 microscope (Leica Microsystems, Wetzlar, Germany). Computer data processing was carried out using Microsoft Excel 2019 MSO version 16.0.13530.20054 (Microsoft Corporation, Redmond, WA, United States) and GraphPad Prism version 9.1.0 (GraphPad Software Inc., San Diego, CA, United States) programs.
Data format	Raw and Analyzed
Description of data collection	Animals were weighed on day 0, then on days 6, 13, 20, 27, 34 and 41 of the experiment. A total of 81 lung tissue samples from Syrian hamsters were obtained for histological examination. Semi-quantitative evaluation of histological preparations was carried out using a scoring system, including the following features: lung inflammation, cell infiltration, pulmonary edema. Each sign was evaluated on the following scale: (0) - absent, (1) - mild, (2) - moderate, (3) - severe. Before the start of the experiment, a uniform test protocol and a data evaluation system were discussed and approved.
Data source location	Department of Virology, Faculty of Biology, Lomonosov Moscow State University, 1–12 Leninskie gory, Moscow 119,234, Russia
Data accessibility	Repository name: Mendeley Data Direct link to the dataset: https://data.mendeley.com/datasets/sx2s29jf95/2
Related research article	A.O. Kovalenko, E.M. Ryabchevskaya, E.A. Evtushenko, T.I. Manukhova, O.A. Kondakova, P.A. Ivanov, M.V. Arkhipenko, V.A. Gushchin, N.A. Nikitin, O.V. Karpova, Vaccine Candidate Against COVID-19 Based on Structurally Modified Plant Virus as an Adjuvant, Front Microbiol. 13 (2022) 845,316. doi:10.3389/fmicb.2022.845316

## Value of the Data

• The present data are useful for further development of a COVID-19 vaccine represented by spherical particles (SPs) derived from tobacco mosaic virus (TMV) in composition with three recombinant coronavirus antigens (3AG), expressed in E. coli bacterial system.
• These data are valuable for the development of human vaccines against COVID-19 and for researchers interested in plant virus-based adjuvants.
• The data could be used for design of further studies focused on the safety and protective efficacy evaluation of COVID-19 vaccine candidates represented by SPs+3AG compositions.

## 1 Objective

The presented data are related to the research article “Vaccine Candidate Against COVID-19 based on Structurally Modified Plant Virus as an Adjuvant” (Kovalenko et al., 2022) on the development of a vaccine candidate against COVID-19 represented by SPs+3AG compositions. The immunogenicity of this vaccine candidate was demonstrated on mice model. Moreover, the sera collected from vaccinated hamsters were shown to possess neutralizing activity against SARS-CoV-2 *in vitro* [1]. Therefore, the objective of the present study was to obtain additional data on the safety and ability to induce protective effect of the SPs+3AG-based vaccine candidate *in vivo*. Here were analyzed two vaccine candidates that differ in the total amount of recombinant coronavirus antigens, MSU-CoV-4 (45 µg) and MSU-CoV-5 (60 µg).

## 2 Data Description

Here we provide data related to MSU-CoV-4 and MSU-CoV-5 vaccine candidates against COVID-19 safety and protective efficacy evaluation.  The safety of the vaccine candidates was evaluated by monitoring the physiological condition of laboratory animals (Syrian hamsters) and their body weight during immunization. The protective efficacy of MSU-CoV-4 and MSU-CoV-5 was investigated by analyzing histological changes in the lungs of vaccinated hamsters after SARS-CoV-2 challenge. Images of the lungs of Syrian hamsters and Table of the body weight raw data are available at https://data.mendeley.com/datasets/sx2s29jf95/2 [2].

The weights (mean±SD) of the experimental animals are shown in Table 1. Throughout the whole period of observation, the animals were in the physiological norm.

**Table 1:** The effect of MSU-CoV-4 and MSU-CoV-5 vaccine candidates on the body weight of Syrian hamsters (*n* = 20).

		Average body weight of the animals in each group on the days of analysis (mean±SD)						
#	Group	0	6	13	20	27	34	41
1	MSU-CoV-4 (SPs (250 μg) + 3AG (45 μg) in PBS)	42.3 ± 0.7	49.1 ± 1.1	49.4 ± 2.3	57.9 ± 4.2	68.5 ± 2.5	73.9 ± 2.1	81.8 ± 4.8
2	MSU-CoV-5 (SPs (250 μg) + 3AG (60 μg) in PBS)	41.2 ± 0.8	49.5 ± 1.7	56.0 ± 2.2	65.6 ± 4.3	75.4 ± 5.4	83.4 ± 5.5	91.9 ± 7.0
3	Placebo (PBS)	41.8 ± 1.4	48.9 ± 1.7	51.3 ± 3.3	57.7 ± 4.8	67.9 ± 5.9	73.5 ± 8.5	78.8 ± 9.2
4	Control of the virus dose	42.9 ± 1.1	51.2 ± 1.5	56.7 ± 2.2	63.6 ± 3.0	73.9 ± 8.5	79.6 ± 10.2	88.4 ± 7.8
5	Intact	43.1 ± 1.2	52.6 ± 1.7	55.9 ± 1.3	65.4 ± 1.6	75.7 ± 2.4	79.8 ± 3.1	91.1 ± 3.0

**Fig. 1:**
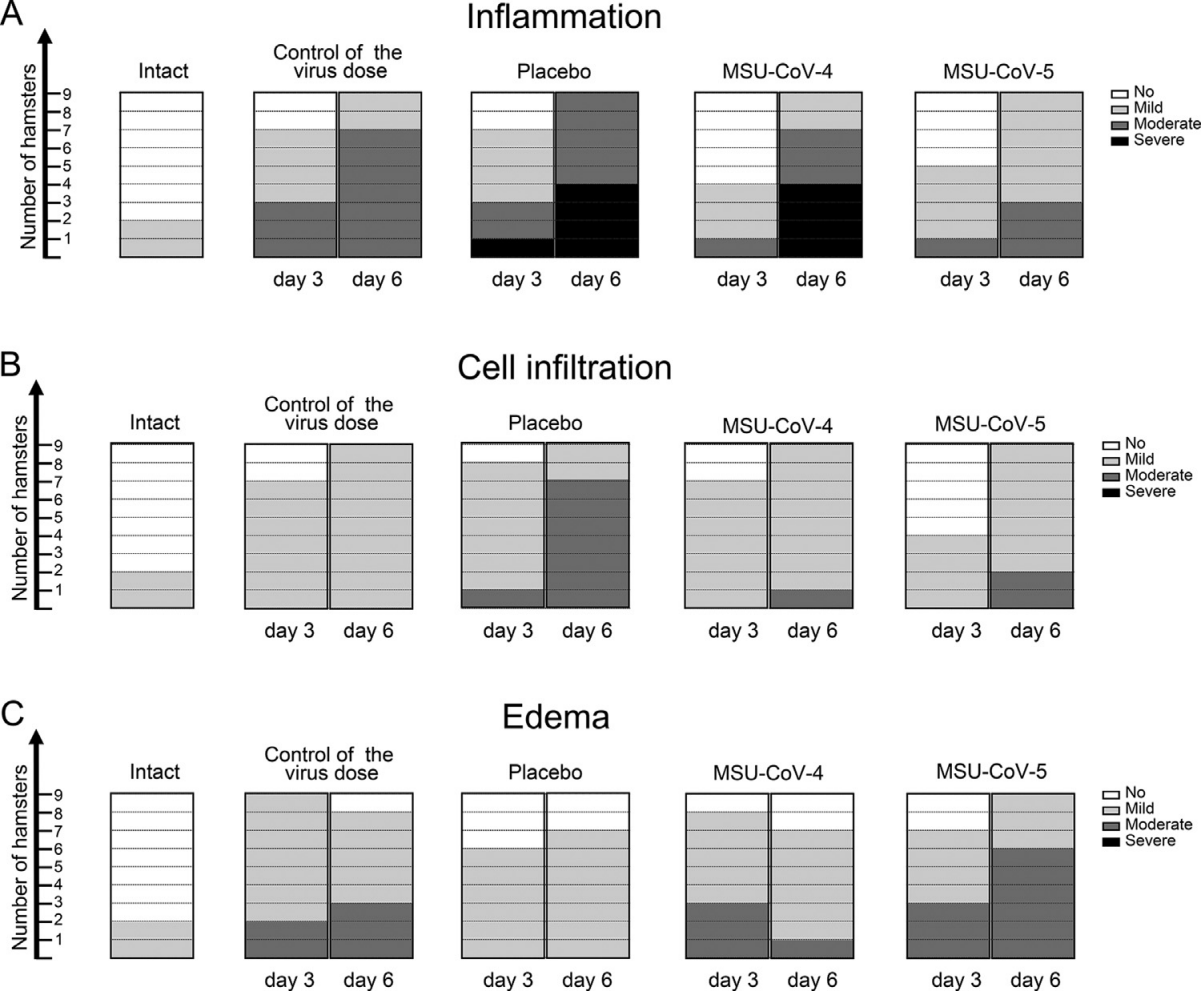
The number of hamsters in each group with mild, moderate or severe lung pathology, which was assessed on signs of lung inflammation (**A**), cell infiltration (**B**) and pulmonary edema (**C**).

Histological examination of the lungs of Syrian hamsters using hematoxylin and eosin staining was carried out (81 samples in total). The specificity and severity of lung pathology were determined. Fig. 1 shows the number of hamsters in each group with mild, moderate or severe lung pathology, which was assessed on the following signs: lung inflammation (A), cell infiltration (B) and pulmonary edema (C).

**Fig. 2:**
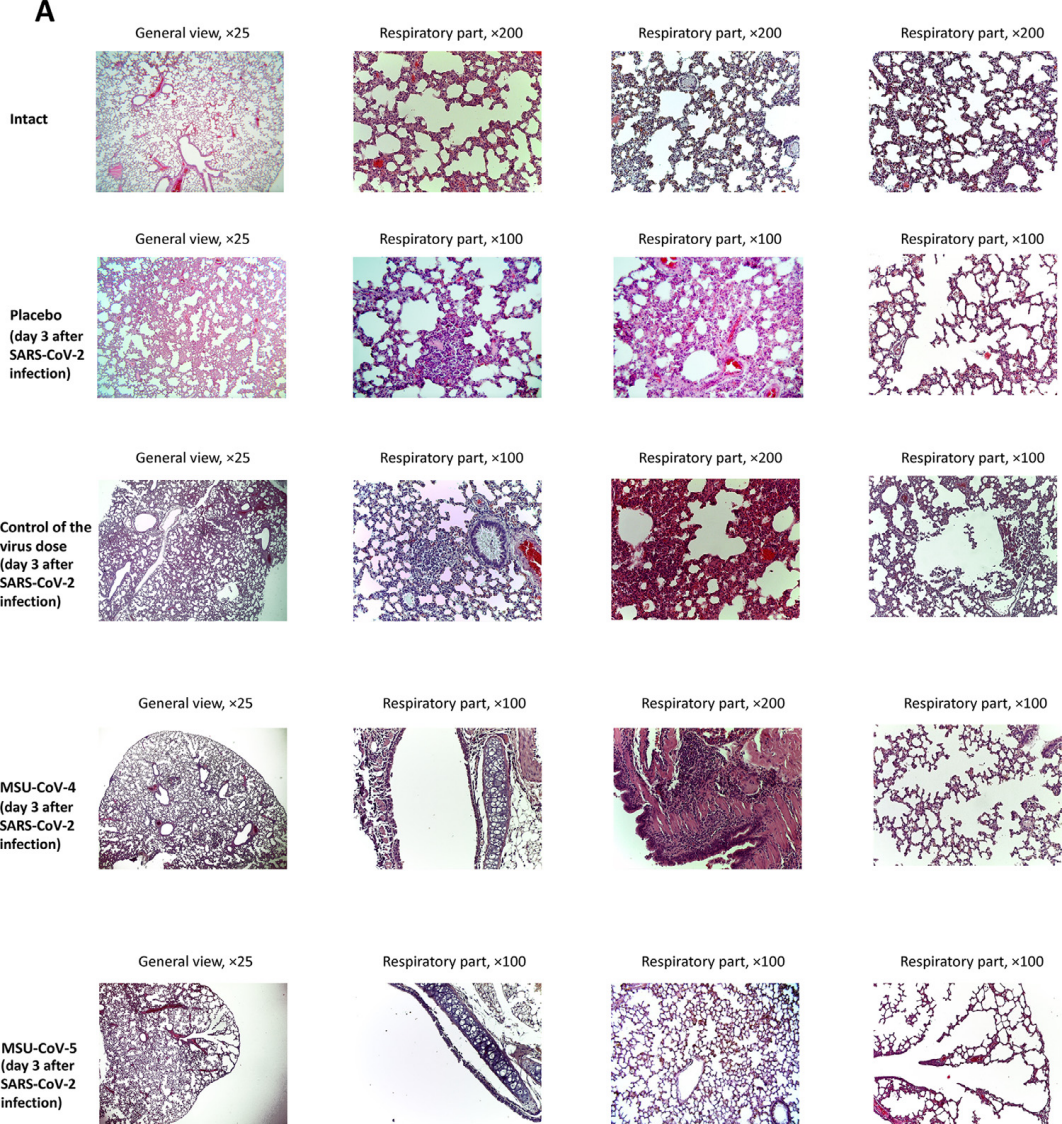

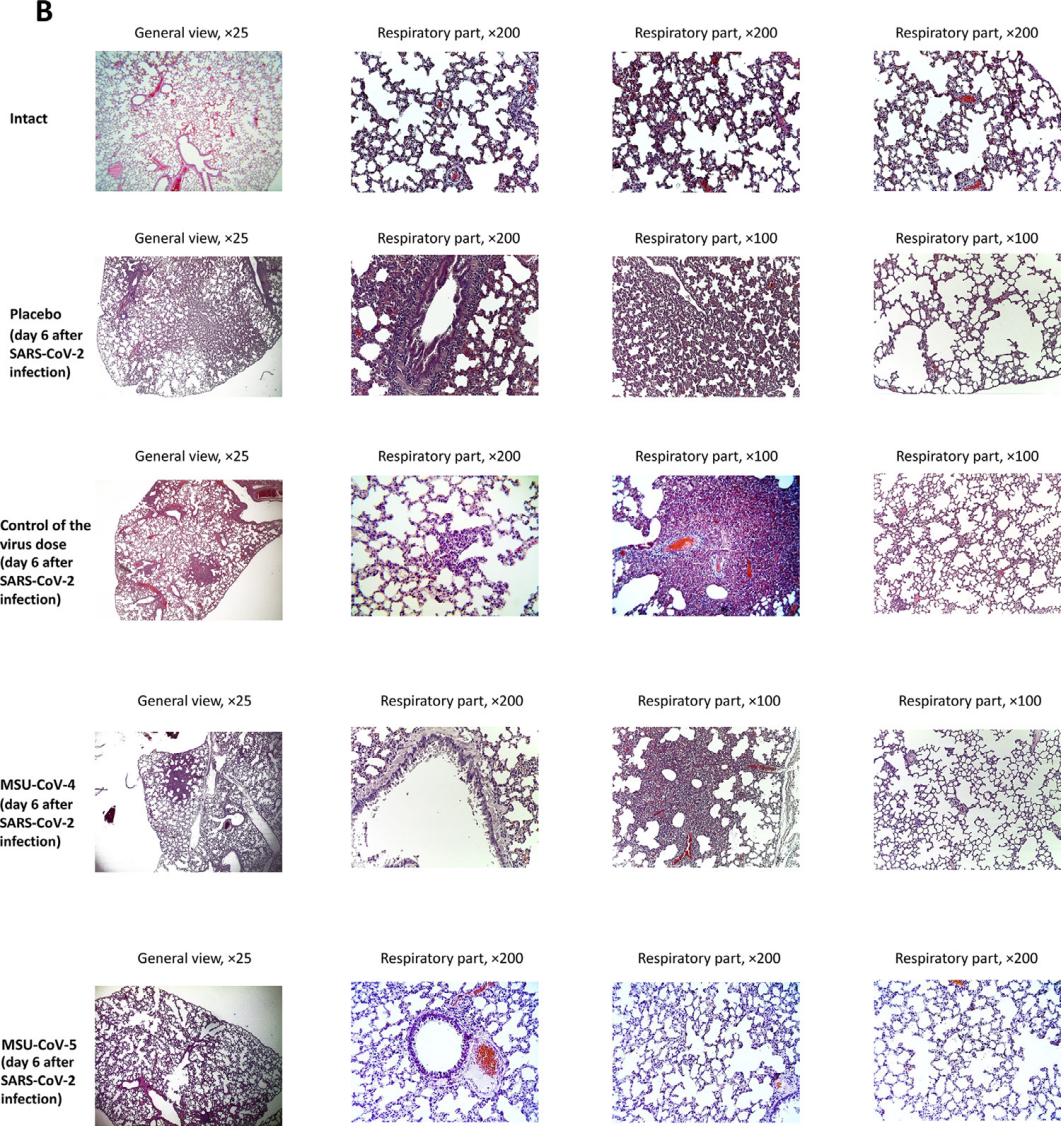
Representative images of the lungs of Syrian hamsters on the day 3 (**A**) and on the day 6 (**B**) after SARS-CoV-2 infection. The lung histological structure of group 5 (intact) corresponded to the norm; no considerable differences in the lung structure of intact animals were found. The detected pathomorphological signs in the lungs of groups 1–4 after SARS-CoV-2 challenge confirmed the presence of the infection process.

Fig. 2 shows representative images of the lungs of Syrian hamsters on the 3rd (A) and 6th (B) days after infection with the SARS-CoV-2 virus.

## 3 Experimental Design, Materials and Methods

### 3.1 Vaccine Candidates

The vaccine candidates against COVID-19 are the compositions (SPs+3AG) of three recombinant coronavirus antigens (3AG) and spherical particles (SPs), formed by thermal treatment of tobacco mosaic virus (TMV). The preparations of TMV and SPs were obtained as described in Trifonova et al. [3]. The SPs samples were analyzed as described in Evtushenko et al. using a JEM-1011 microscope (JEOL, Akishima, Tokyo, Japan) [4]. The scientific image manipulation software ImageJ (National Institutes of Health, United States) was used to calculate the size. The mean diameter of SPs was 389±92 (mean±SD, *n* = 100).

The following recombinant proteins were expressed in *E. coli* and used as vaccine antigens [1]: Co1 – contains a sequence of receptor-binding domain (RBD) (residues 319–541) of SARS-CoV-2 S-protein; PE (polyepitope protein) – contains epitopes of the S2-subunit of S-protein, which are highly conserved for SARS-CoV-2, SARS-CoV and other SARS-like betacoronaviruses; CoF – contains the RBD sequence of the SARS-CoV-2 S-protein and S2-subunit epitope [residues 1182–1210 in the coordinates of the reference sequence (Wuhan-Hu-1, GenBank YP_009724390.1)].

MSU-CoV-4 and MSU-CoV-5 vaccine candidates were prepared by mixing of recombinant antigens and SPs in 1x PBS buffer (1.06 mM KH_2_PO_4_, 155.17 mM NaCl, 2.97 mM Na_2_HPO_4_–7H_2_O, pH 7.4) (Cat# 70011-036, Gibco™, Thermo Fisher Scientific, Waltham, Massachusetts, United States). The same buffer was used for preparation of placebo formulation.

The formulation of vaccine candidates:

MSU−CoV−4(15μgPE+15μgCo1+15μgCoF+250μgSPsin0.5mlPBS),

MSU−CoV−5(20μgPE+20μgCo1+20μgCoF+250μgSPsin0.5mlPBS),

The formulation of placebo: 1x PBS (pH 7.4).

**Fig. 3:**
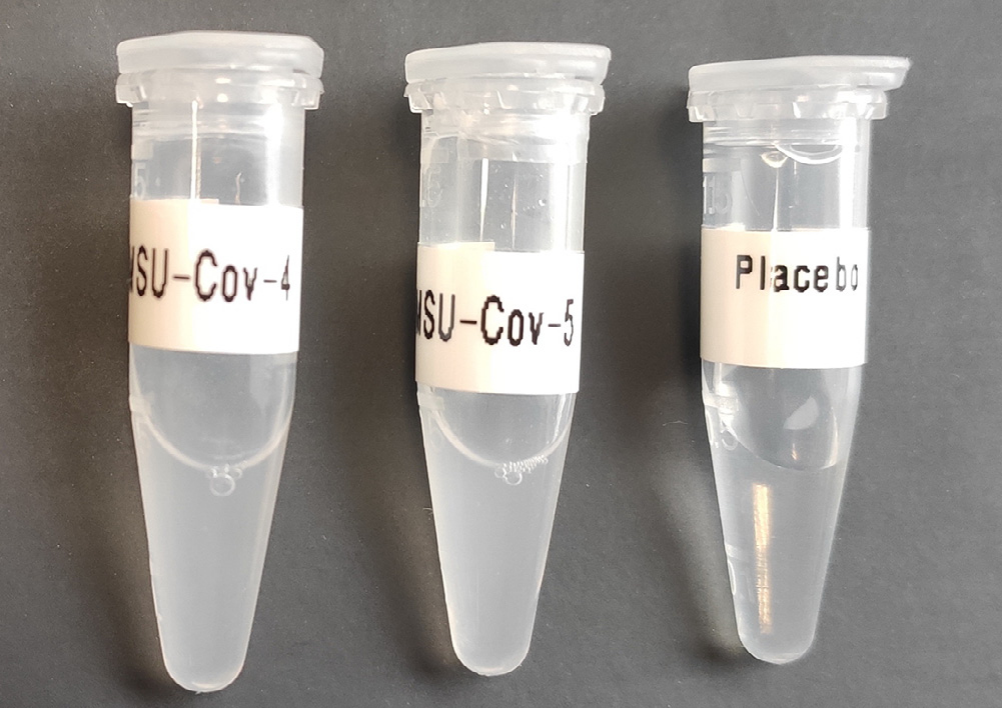
The image of MSU-CoV-4 and MSU-CoV-5 vaccine candidates’ suspensions in comparison to PBS buffer (placebo).

The vaccine candidates are suspensions for intramuscular administration (Fig. 3).

### 3.2 Animals

Syrian hamsters are the optimal model for studying vaccines against COVID-19. According to Rosenke et al. (2020), the sex of animals does not play a significant role in the severity of SARS-CoV-2 infection [5]. In contrast, in a more recent study performed by Dhakal et al. differences in the course of SARS-CoV-2 infection in male and female hamsters were reported [6]. Sex differences in COVID-19 outcomes in humans are also described [7,8]. Therefore, there is a limitation of our study due to the fact that it was performed only on female Syrian hamsters. The golden Syrian hamsters (Mesocricetus auratus) (female; weight of 40–45 g) obtained from the Branch of the Shemyakin–Ovchinnikov Institute of Bioorganic Chemistry of the Russian Academy of Sciences (Nursery for the Laboratory Animals, Pushchino, Russia) were used.

### 3.3 SARS-CoV-2 Strain

The SARS-CoV-2 Wuhan strain was isolated from the nasopharyngeal aspirate specimen of a patient with COVID-19. All experiments with live SARS-CoV-2 were performed at a biosafety level-3 (BSL-3) facility.

### 3.4 Design of Safety and Protective Efficacy Study of Vaccine Candidates

Before the start of the experiment, the animals received from the breeder were acclimatized for three days. Before immunization, the Syrian hamsters’ behavior, appetite, coat and mucous membranes were examined. Five experimental groups of animals were formed to study the efficacy of the MSU-CoV-4 and MSU-CoV-5 vaccine candidates. Each group included 20 hamsters. Groups 1 and 2 were immunized with MSU-CoV-4 and MSU-CoV-5 vaccine candidates, respectively. Group 3 (placebo) were injected with the same volume of PBS buffer. The formulations were administered to animals in groups 1–3 on the 1st and 22nd day of the experiment intramuscularly, in volume 0.5 ml per animal. Fractional injections of 0.25 ml (in two pelvic limbs in equal volumes) with an interval of 1 hour were used. Animals were weighed on day 0 (before the experiment), then on days 6, 13, 20, 27, 34 and 41 of the experiment. When weighing, four animals were randomly selected from each group to determine body weight (five weightings per group).  The physiological condition (behavior, motor activity, appetite, fatness, coat, skin, etc.) of Syrian hamsters was monitored daily for compliance with the norm.

On the 43rd day of the experiment, animals were infected intranasally with SARS-CoV-2 virus at a dose of 5.0 lg PFU. In group 4 (control of the virus dose) animals were infected with SARS-CoV-2 virus without prior immunization. In group 5 (intact), immunization and infection manipulations were not carried out with the animals. For protective efficacy evaluation, 18 animals from each group (groups 1–4) and nine animals from the group 5 (intact) were used, the rest of the animals was not used in the study. In three days after infection (day 46 of the experiment), in groups 1–4, nine animals were euthanized and the remaining nine animals were euthanized in six days after challenge (day 49 of the experiment). In group 5 (intact), three animals were euthanized on day 46 of the experiment and six animals on day 49 of the experiment. Euthanasia was performed by cervical dislocation. After the necropsy of Syrian hamsters, the size, shape and position of the internal organs were assessed for compliance with the norm.

**Fig. 4:**
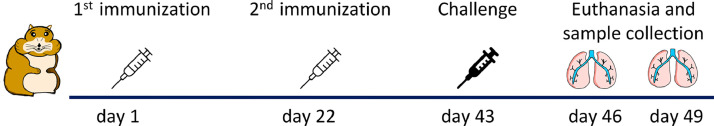
Scheme of MSU-CoV-4 and MSU-CoV-5 protective efficacy analysis in a model of SARS-CoV-2 infection in Syrian hamsters.

The design of the study of the protective efficacy against COVID-19 in Syrian hamsters is shown in Fig. 4 and the group description is presented in Table 2.

**Table 2:** The design of protective efficacy analysis of vaccine candidates (MSU-CoV-4 and MSU-CoV-5) against COVID-19 in Syrian hamsters.

					Euthanasia (days and number of animals)	

#	Group	Single dose volume (ml)	Days and route of vaccine candidates’ administration	Infection with the SARS-CoV-2	day 46	day 49
1	MSU-CoV-4 (SPs (250 μg) + 3AG (45 μg) in PBS)	0.5	Intramuscularly, on days 1 and 22 of the experiment	On day 43 of the experiment, intranasally at a dose of 5.0 lg PFU	9	–
					–	9
2	MSU-CoV-5 (SPs (250 μg) + 3AG (60 μg) in PBS)	0.5			9	–
					–	9
3	Placebo (PBS)	0.5			9	–
					–	9
4	Control of the virus dose	–	–		9	–
					–	9
5	Intact	–	–	–	3	–
					–	6

After euthanasia, one separated lung from each animal was placed in a 10% formalin buffered solution for histological examination (a total of 81 Syrian hamsters’ lung tissue samples were obtained in the study).

### 3.5 Lung Histological Studies of Syrian Hamsters

Histopathological changes in hamster lungs after SARS-CoV-2 infection were assessed by hematoxylin and eosin staining. Lung tissues were first fixed in 10% buffered formalin, and then processed for paraffin embedding. Paraffin blocks were cut into 2-µm sections and stained with hematoxylin and eosin. Histological preparations were examined, assessing such signs of lung pathology as inflammation, cell infiltration by neutrophils, macrophages and lymphocytes, and pulmonary edema. Microscopic lung injury was classified into 3 types: mild, moderate and severe.

### 3.6 Data Processing

Computer data processing was carried out using Microsoft Excel 2019 MSO version 16.0.13530.20054 (Microsoft Corporation, Redmond, WA, United States) and GraphPad Prism version 9.1.0 (GraphPad Software Inc., San Diego, CA, United States) programs.

## Ethics Statements

Animal studies were performed under protocols approved by the Federal State Budgetary Institution, Central Research Institute No. 48 of the Russian Ministry of Defense (Sergiev Posad, Russia) (protocol №15 dated 13.12.2021), in accordance by the Decision of Council of the Eurasian economic commission of November 3, 2016 No. 81 “Rules of proper laboratory practice of the Eurasian Economic Union in the field of drug circulation”, U.S. FDA Good Laboratory Practice (GLP) Regulations for Non-clinical Laboratory Studies (21 CFR Part 58) and OECD Principles of Good Laboratory Practice (ENV/MC/CHEM (98) 17).

## CRediT authorship contribution statement

**A.O. Kovalenko:** Conceptualization, Methodology, Validation, Investigation, Writing – original draft. **E.M. Ryabchevskaya:** Conceptualization, Methodology, Investigation, Writing – review & editing, Visualization. **E.A. Evtushenko:** Conceptualization, Methodology, Validation, Investigation, Writing – review & editing. **O.A. Kondakova:** Conceptualization, Writing – review & editing. **P.A. Ivanov:** Methodology, Investigation, Writing – review & editing. **M.V. Arkhipenko:** Methodology, Investigation, Writing – review & editing. **N.A. Nikitin:** Conceptualization, Validation, Investigation, Writing – review & editing, Project administration. **O.V. Karpova:** Conceptualization, Resources, Writing – review & editing, Supervision, Funding acquisition.

## Declaration of Competing Interest

The authors declare that they have no known competing financial interests or personal relationships that could have appeared to influence the work reported in this paper.

## Data Availability

Images of the lungs of Syrian hamsters and Table of the body weight raw data (Original data) (Mendeley Data).
